# A Young Boy with Fever and Grunting

**DOI:** 10.5811/cpcem.2020.11.49721

**Published:** 2021-01-16

**Authors:** Daniel J. Shapiro, Jeffrey T. Neal

**Affiliations:** Boston Children’s Hospital, Department of Pediatrics, Boston, Massachusetts. Boston Children’s Hospital, Division of Emergency Medicine, Boston, Massachusetts

**Keywords:** pneumoperitoneum, grunting, pediatric abdominal emergencies

## Abstract

**Case Presentation:**

A 16-month-old boy presented with a temperature of 99°Fahrenheit (F) (down from 102°F at home after antipyretics), grunting, and tachypnea. On examination, he was tachycardic, tachypneic, and ill-appearing with abdominal distention and diffuse tenderness. A plain film abdominal radiograph showed moderate free air, and emergent laparoscopy revealed perforated Meckel’s diverticulitis with peritonitis.

**Discussion:**

Although tachypnea and grunting in preverbal or nonverbal patients are often considered to be signs of respiratory illness, these findings may reflect intra-abdominal emergencies. Perforated Meckel’s diverticulitis is an important differential consideration in patients with pneumoperitoneum.

## CASE PRESENTATION

A 16-month-old healthy boy presented to the emergency department with a temperature of 99°Fahrenheit (F) (down from 102°F at home after antipyretics), tachypnea, and grunting. Physical examination demonstrated an ill-appearing child with tachycardia and tachypnea, clear lung fields, and a distended, tender abdomen. Plain film abdominal radiography showed mildly prominent, gas-filled loops of small bowel and moderate free air (Image).

The patient received piperacillin-tazobactam and was transferred to the operating room for exploratory laparoscopy, which revealed an acutely inflamed and bleeding Meckel’s diverticulum with a well-circumscribed perforation. Uneventful diverticulectomy was performed, and the patient was discharged from the hospital five days later after a full recovery.

## DISCUSSION

Although tachypnea and grunting in a preverbal or nonverbal patient may be signs of respiratory illness, abdominal emergencies may present with similar findings.^1^ In such cases, these respiratory phenomena may be physiologic responses to pain or acidemia or may reflect the presence of mechanical thoracoabdominal competition. When an abdominal emergency is suspected in the setting of a respiratory complaint, plain films of the abdomen can provide a rapid assessment for pneumoperitoneum. In this scenario, left lateral decubitus films may be particularly helpful in toxic-appearing young children, in whom obtaining upright radiographs may be logistically challenging.

Meckel’s diverticulum occurs in the mid-to-distal ileum as a remnant of the fetal omphalomesenteric duct. Although present in approximately 2% of the general population, it causes symptoms in less than 10% of cases.^2^,^3^ Symptomatic cases, which occur more commonly in children than in adults, most often present with painless bleeding from heterotopic gastric tissue.^4^ However, Meckel’s diverticulum may cause small bowel obstruction by (1) acting as a lead point for intussusception; (2) inverting into the bowel lumen; or (3) adhering to adjacent structures to cause a volvulus.^5^ Similarly, Meckel’s diverticulitis may cause acute abdominal pain that mimics appendicitis and can subsequently perforate to cause peritonitis. Accordingly, Meckel’s diverticulum is an important differential consideration in the acute surgical abdomen, particularly in pediatric patients.

**CPC-EM Capsule**

What do we already know about this clinical entity?

Tachypnea and grunting are often considered signs of respiratory illness, but intra-abdominal emergencies may present with similar findings.

What is the major impact of the image(s)?

These images provide an example of an intra-abdominal emergency presenting with primarily respiratory symptoms and signs.

How might this improve emergency medicine practice?

These images emphasize the importance of considering alternative causes of respiratory symptoms and signs, particularly in nonverbal or preverbal patients.

## Figures and Tables

**Image:**
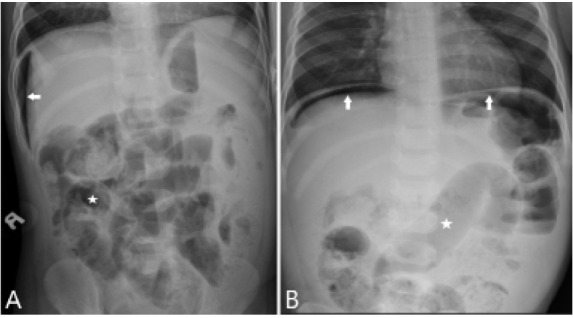
Plain film abdominal radiographs. Panel A (left lateral decubitus view) and Panel B (upright anteroposterior view) showing free air under the diaphragm (arrows) with dilated loops of bowel (stars).
